# Dynamic change of heart rate in the acute phase and clinical outcomes after intracerebral hemorrhage: a cohort study

**DOI:** 10.1186/s40560-021-00540-0

**Published:** 2021-03-18

**Authors:** Shoujiang You, Yupin Wang, Zian Lu, Dandan Chu, Qiao Han, Jiaping Xu, Chun-Feng Liu, Yongjun Cao, Chongke Zhong

**Affiliations:** 1 Department of Neurology and Suzhou Clinical Research Center of Neurological Disease, The Second Affiliated Hospital of Soochow University, No. 1055 Sanxiang Road, Suzhou, 215004 Jiangsu China; 2 Department of Epidemiology, School of Public Health and Jiangsu Key Laboratory of Preventive and Translational Medicine for Geriatric Diseases, Medical College of Soochow University, 199 Renai Road, Industrial Park District, Suzhou, 215123 Jiangsu China; 3 Department of Neurology, The People’s Hospital of Xuan Cheng City, Xuancheng, China; 4 Department of Neurology, Suzhou TCM Hospital Affiliated to Nanjing University of Chinese Medicine, Suzhou, China; 5 Institutes of Neuroscience, Soochow University, Suzhou, China

**Keywords:** Heart rate, Dynamic change, Trajectory, Variability, Functional outcome, Mortality, Acute intracerebral hemorrhage

## Abstract

**Background:**

Dynamic change of heart rate in the acute phase and clinical outcomes after intracerebral hemorrhage (ICH) remains unknown. We aimed to investigate the associations of heart rate trajectories and variability with functional outcome and mortality in patients with acute ICH.

**Methods:**

This prospective study was conducted among 332 patients with acute ICH. Latent mixture modeling was used to identify heart rate trajectories during the first 72 h of hospitalization after ICH onset. Mean and coefficient of variation of heart rate measurements were calculated. The study outcomes included unfavorable functional outcome, ordinal shift of modified Rankin Scale score, and all-cause mortality.

**Results:**

We identified 3 distinct heart rate trajectory patterns (persistent-high, moderate-stable, and low-stable). During 3-month follow-up, 103 (31.0%) patients had unfavorable functional outcome and 46 (13.9%) patients died. In multivariable-adjusted model, compared with patients in low-stable trajectory, patients in persistent-high trajectory had the highest odds of poor functional outcome (odds ratio 15.06, 95% CI 3.67–61.78). Higher mean and coefficient of variation of heart rate were also associated with increased risk of unfavorable functional outcome (*P* trend < 0.05), and the corresponding odds ratios (95% CI) comparing two extreme tertiles were 4.69 (2.04–10.75) and 2.43 (1.09–5.39), respectively. Likewise, similar prognostic effects of heart rate dynamic changes on high modified Rankin Scale score and all-cause mortality were observed.

**Conclusions:**

Persistently high heart rate and higher variability in the acute phase were associated with increased risk of unfavorable functional outcome in patients with acute ICH.

**Supplementary Information:**

The online version contains supplementary material available at 10.1186/s40560-021-00540-0.

## Background

Intracerebral hemorrhage (ICH) accounts for 10–15% of all strokes and is the most serious type of stroke with high mortality [1]. It is extremely important to identify and manage risk factors associated with clinical outcomes after ICH. Heart rate is an accessible and useful clinical indicator of autonomic balance. Numerous epidemiological studies have reported that single measurement of resting heart rate is an independent predictor of stroke and all-cause mortality in the general population [2–5]. Moreover, there is also evidence supporting resting heart rate as a prognostic risk factor in several cardiovascular conditions, including hypertension, traumatic brain injury, and heart failure [6–9]. Furthermore, autonomic dysfunction or elevated heart rate is commonly observed after acute stroke and was suggested to be associated with adverse clinical outcomes in patients with acute ischemic stroke [10–14]. However, evidence on the associations between heart rate and clinical outcomes in acute ICH patients is rare [15].

Dynamic change of heart rate after acute stroke is common. However, the majority of studies on heart rate and prognosis of acute stroke were based on a single measure of heart rate, failing to take into account the potential effect of heart rate change. To our knowledge, there is no study that had examined the trajectories of heart rate change and clinical outcomes of patients with acute ICH. We hypothesized that dynamic change of heart rate in the acute phase of ICH may provide additional prognostic information. Accordingly, we aimed to identify subgroups of patients with similar trajectories of heart rate change during the acute phase and to investigate the associations between heart rate trajectories with functional outcome and mortality in patients with acute ICH. Additionally, we also assessed the predictive value of heart rate variability on clinical outcomes.

## Methods

### Study participants

This prospective study was conducted in acute ICH patients recruited from the Second Affiliated Hospital of Soochow University in China, during November 2011 to March 2014. The detailed methods for the recruitment of study participants have been described elsewhere [16, 17]. In brief, a total of 413 eligible patients aged ≥ 18 years who had acute ICH confirmed by computed tomography were recruited. All the patients were enrolled from the Department of Neurology without neurosurgical treatment based on the baseline hematoma volume and location at emergency department. Patients with trauma, brain tumor, hemorrhagic transformation of ischemic stroke, and vascular cerebral malformations were excluded. In the present study, the additional exclusion criteria were as follows: (1) requirement for neurosurgical procedures (*n* = 10); (2) time from onset to admission over 7 days (*n* = 11); (3) < 3 heart rate measurements during the first 72 h of hospitalization (*n* = 33). After further excluded patients without 3-month modified Rankin Scale score (*n* = 27), 332 patients were finally included in this analysis (Supplemental Figure 1).

This study was approved by the Ethics Committee of the Second Affiliated Hospital of Soochow University, and informed consent was obtained from all participants.

### Data collection

Data on demographic characteristics, clinical features, medical history (hypertension, diabetes mellitus, stroke, and atrial fibrillation), imaging data, and time from onset to admission were collected at the time of enrollment. All information was obtained using a standard questionnaire that was administered by trained staff. Stroke severity was assessed using the National Institutes of Health Stroke Scale (NIHSS) by trained neurologists at admission [18]. Hematoma volumes were calculated using the ABC/2 formula by two neuroradiologists who were blinded to the baseline characteristics of study participants [19]. Three blood pressure (BP) measurements were also obtained at admission by trained nurses using a standard mercury sphygmomanometer according to a standard protocol. Antihypertensive medication use during hospitalization was collected, and types of antihypertensive medications included calcium channel blocker, beta-blocker, angiotensin-converting enzyme inhibitor/angiotensin II receptor blockers, diuretic, and the combination of 2 or more drugs (Supplemental Table 1). Routine laboratory determinations (plasma glucose, blood lipids, etc.) were performed for all enrolled patients using standard procedures within 24 h after admission. In this study, heart rate was automatically measured from an electrocardiogram monitor. Serial heart rate measurements were recorded at baseline, every 6 h in the first 24 h after enrollment, and at 48 and 72 h. Therefore, a total of 7 heart rate measurements were used to fit heart rate trajectories. In addition, the mean and coefficient of variation (CV) of the 7 heart rate measurements were calculated.

### Outcome assessment

The participants were followed up in person at 3 months after ICH by trained neurologists. The study outcome was evaluated using the modified Rankin Scale (scores range from 0, indicating no symptoms, to 6, indicating death). The primary outcome was unfavorable functional outcome of severe disability or death, defined as a modified Rankin Scale score of 4–6. The secondary outcomes included an ordinal shift in modified Rankin Scale score and 3-month mortality from all causes. Deaths were reported by their relatives, work associates in-person or telephone interviews, and/or obtained from death certificates and medical records at 3 months.

### Statistical analysis

**Fig. 1:**
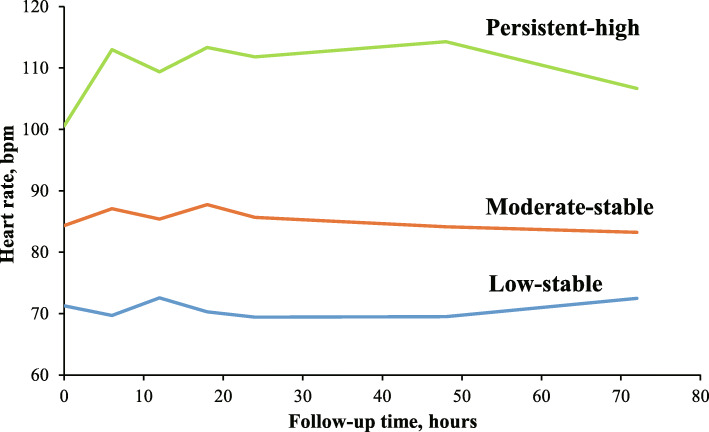
Trajectories of heart rate during the acute phase of intracerebral hemorrhage

Latent mixture modeling was used to identify subgroups that share similar underlying trajectories of heart rate during the first 72 h of hospitalization after ICH onset. The method was implemented using SAS PROC TRAJ [20, 21]. The best-fitting trajectory models were assessed according to the Bayesian information criterion (BIC). We used a two-stage approach to select the optimal number of groups and the shapes of trajectories. First, we fitted all trajectory models in cubic form and initiated a model with 1 trajectory and then fitted models up to the optimal number of trajectories according to the comparison of BIC for each set of trajectories. The minimum sample size of each trajectory was specified to be more than 5%. Given the data we had, we chose to use 3 subgroups, the model with 3 trajectories identified fitted best, and increasing the number of trajectory groups led to small group sizes. In the second stage, we compared the model fit with different functional forms based on their significance level, starting with the highest polynomial. In our final model, we had one pattern with a linear order term and two patterns with up to cubic order terms. We named these 3 trajectories on the basis of the visual patterns of dynamic change of heart rate: persistent-high, moderate-stable, and low-stable (Fig. 1). We then calculated the posterior predicted probability for each individual of being a member of each of the 3 trajectories. Each membership was assigned to the trajectory to which he or she holds the highest posterior membership probability, and the average posterior probability for each trajectory group was 0.98, 0.90, and 0.90 for persistent-high, moderate-stable, and low-stable group, respectively. The distribution of posterior predicted probability is presented in Supplemental Figure 2.

In addition, study participants were also categorized into 3 subgroups according to tertile of mean or CV of heart rate. Categorical and ordinal logistic regression models were used to examine the associations between trajectories, mean, and CV of heart rate with functional outcome and modified Rankin Scale score. The cumulative risks of 3-month all-cause mortality were estimated with Kaplan-Meier curves and compared using the log-rank test. Cox proportional hazards regression was used to estimate the risk of all-cause mortality. Odds ratios (ORs) or hazard ratios (HRs) and 95% confidence intervals (CIs) were calculated for each subgroup with the low-stable trajectory or lowest tertile as reference group. Potential covariates, such as age, sex, time from onset to admission, current smoking status, systolic BP, fasting plasma glucose, high-density lipoprotein cholesterol, medical history (hypertension, diabetes mellitus, stroke, and atrial fibrillation), types of antihypertensive medications, baseline NIHSS score, location, and volume of hematoma, were included in the multivariable models. Assessment for linear trend was performed by setting heart rate subgroups as ordinal variables in the models. Multiple imputation for missing data of covariates was performed using the Markov chain Monte Carlo method. All *P* values were 2-tailed, and a significance level of 0.05 was used. Statistical analysis was conducted using SAS statistical software (version 9.4, Cary, NC, USA).

## Results

### Baseline characteristics

There were 332 eligible acute ICH patients (224 men and 108 women) included in our study with a mean age of 64.3 ± 13.7 years. The median of NIHSS score and time from onset to admission were 8 (interquartile range 3–13) and 5 h (interquartile range 3–24 h), separately. Baseline characteristics of study participants are shown in Table 1. Compared with participants in low-stable trajectory group, those in persistent-high group were more likely to be older and had higher admission systolic and diastolic BP, blood lipids, fasting plasma glucose levels, admission NIHSS score, and hematoma volume, and had higher proportion of intraventricular extension. During 3 months of follow-up, a total of 103 (31.0%) patients had poor functional outcome and 46 (13.9%) patients died from all causes. Compared with study participants with good functional outcome, those with poor outcome were older, had higher high-density lipoprotein cholesterol, plasma glucose, admission NIHSS score, and hematoma volume, and had higher prevalence of history of stroke, while had lower time from onset to admission and prevalence of use of antihypertensive medication in the first 24 h (Supplemental Table 2).

**Table 1:** Baseline characteristics of patients with acute intracerebral hemorrhage according to the trajectory of heart rate

Characteristics^a^	Total population (*n* = 332)	Trajectory of heart rate			*P* value
		Group 1: low-stable (*n* = 174)	Group 2: moderate-stable (*n* = 126)	Group 3: persistent-high (*n* = 32)	
Demographics					
Age, years	64.3 ± 13.7	64.1 ± 13.7	63.0 ± 13.6	69.9 ± 13.0	0.04
Male	224 (67.5)	119 (68.4)	83 (65.9)	22 (68.8)	0.89
Current smoking	69 (20.8)	38 (21.8)	26 (20.6)	5 (15.6)	0.73
Clinical features					
Time from onset to admission, h	5.0 (3.0-24.0)	6.0 (3.0-24.0)	5.0 (3.0-12.0)	6.0 (3.0-16.0)	0.32
Systolic BP, mmHg	169.2 ± 28.9	162.3 ± 27.2	175.7 ± 28.0	181.0 ± 32.6	< 0.001
Diastolic BP, mmHg	94.9 ± 16.7	89.9 ± 14.5	100.3 ± 17.2	100.7 ± 17.6	< 0.001
Triglyceride, mmol/L	1.0 (0.8–1.4)	0.9 (0.7–1.3)	1.2 (0.8–1.8)	1.1 (0.9–1.6)	0.005
Total cholesterol, mmol/L	4.6 (4.1–5.4)	4.5 (4.0–5.2)	4.8 (4.2–5.6)	4.8 (4.2–5.9)	0.01
LDL cholesterol, mmol/L	2.8 (2.3–3.4)	2.7 (2.2–3.2)	2.9 (2.3–3.7)	2.6 (2.1–3.6)	0.03
HDL cholesterol, mmol/L	1.3 (1.1–1.6)	1.3 (1.1–1.5)	1.2 (1.0–1.6)	1.5 (1.2–1.8)	0.01
Fasting plasma glucose, mol/L	5.9 (5.2–7.0)	5.7 (5.2–6.5)	6.1 (5.3–7.3)	8.5 (6.5–10.2)	< 0.001
Baseline NIHSS score	8 (3–13)	5 (3–11)	9 (4–13)	24 (15–25)	< 0.001
Medical history					
History of hypertension	275 (82.8)	136 (79.9)	111 (88.1)	25 (78.1)	0.13
History of diabetes mellitus	41 (12.4)	16 (9.2)	20 (15.9)	5 (15.6)	0.19
History of stroke	62 (18.7)	27 (15.5)	29 (23.0)	6 (18.8)	0.26
History of atrial fibrillation	9 (2.7)	2 (1.2)	5 (4.0)	2 (6.3)	0.14
Antihypertensive medications	220 (66.3)	102 (58.6)	97 (77.0)	21 (65.6)	0.004
CT findings					
Hematoma volume (mL)	10.9 (5.0–23.8)	10.0 (4.8–20.0)	11.4 (5.0–26.4)	36.2 (10.8–80.1)	< 0.001
Hematoma location					
Lobar	47 (14.2)	29 (16.7)	15 (11.9)	3 (9.4)	0.36
Basal ganglia	161 (48.5)	85 (48.9)	65 (51.6)	11 (34.4)	0.22
Thalamus	21 (6.3)	10 (5.8)	11 (8.7)	0 (0.0)	0.17
Cerebellum	12 (3.6)	7 (4.0)	5 (4.0)	0 (0.0)	0.51
Brain stem	28 (8.4)	14 (8.1)	8 (6.4)	6 (18.8)	0.08
Intraventricular extension	63 (19.0)	29 (16.7)	22 (17.5)	12 (37.5)	0.02
Mean heart rate, bpm	77 (72–86)	72 (68–76)	84 (81–88)	108 (103–120)	< 0.001
CV of heart rate, bpm	10.1 (7.4–13.9)	10.1 (7.4–13.2)	9.9 (6.8–12.7)	12.8 (9.0–16.1)	0.02

*BP*, blood pressure; *LDL*, low-density lipoprotein; *HDL*, high-density lipoprotein; *NIHSS*, National Institute of Health Stroke Scale; *CV*, coefficient of variation

^a^Continuous variables are expressed as mean ± standard deviation or median (interquartile range). Categorical variables are expressed as number (%)

### Dynamic change of heart rate and clinical outcomes

The event rates of study outcomes were significantly higher among patients in persistent-high trajectory group (Table 2). Patients in persistent-high group had the highest risks of unfavorable functional outcome (*P* for trend < 0.001). In multivariable model 1 of demographic characteristics and clinical features, compared with patients in low-stable group, the ORs (95% CIs) for primary outcome were 1.96 (1.03–3.74) and 15.21 (4.27–54.11) among patients in moderate-stable and persistent-high trajectories, respectively. After further adjusted for medical history, medication treatment, and imaging data, patients in the persistent-high trajectory still had the highest odds of poor functional outcome (OR 15.06, 95% CI 3.67–61.78, *P* for trend < 0.001). In addition, higher mean and CV of heart rate were associated with increased risk of the primary outcome (both *P* for trend < 0.05). Adjusted ORs comparing two extreme tertiles were 4.69 (95% CI 2.04–10.75) for mean heart rate and 2.43 (1.09–5.39) for CV of heart rate in multivariable model 2, respectively.

**Table 2:** Dynamic changes of heart rate in the acute phase and poor functional outcome in patients with acute intracerebral hemorrhage

	Events (%)	Multivariable model 1		Multivariable model 2	
		OR (95% CI)	*P* trend	OR (95% CI)	*P* trend
Trajectory of heart rate					
Group 1	36 (20.7)	1.00	< 0.001	1.00	< 0.001
Group 2	40 (31.8)	1.96 (1.03–3.74)		1.93 (0.95–3.91)	
Group 3	27 (84.4)	15.21 (4.27–54.11)		15.06 (3.67–61.78)	
Mean heart rate					
Tertile 1	19 (18.1)	1.00	< 0.001	1.00	< 0.001
Tertile 2	25 (21.9)	1.32 (0.61–2.86)		1.54 (0.69–3.46)	
Tertile 3	59 (52.2)	4.49 (2.09–9.66)		4.69 (2.04–10.75)	
CV of heart rate					
Tertile 1	20 (17.4)	1.00	0.006	1.00	0.04
Tertile 2	37 (34.3)	2.52 (1.15–5.50)		2.46 (1.09–5.52)	
Tertile 3	46 (42.2)	2.96 (1.40–6.29)		2.43 (1.09–5.39)	

Multivariable model 1 included age, sex, time from onset to admission, current smoking status, systolic BP, fasting plasma glucose, high-density lipoprotein cholesterol, and baseline NIHSS score. Multivariable model 2 further included medical history (hypertension, diabetes mellitus, stroke, and atrial fibrillation), types of antihypertensive medications, location and volume of hematoma

*OR*, odds ratio; *CI*, confidence interval; *CV*, coefficient of variation

**Fig. 2:**
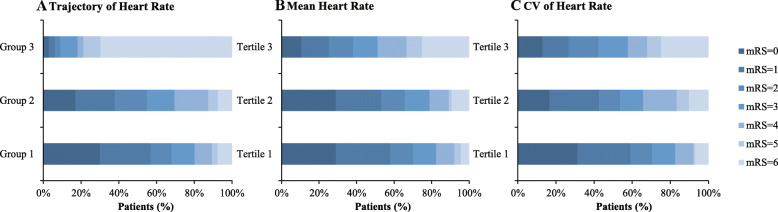
Distribution of modified Rankin Scale score. Patients in persistent-high trajectory: highest tertile of mean and CV had higher modified Rankin Scale score (all *P* < 0.001). **a** Trajectory of heart rate. **b** Mean heart rate. **c** CV of heart rate

Patients in the persistent-high group had higher modified Rankin Scale score (*P* < 0.001), and the medians (interquartile ranges) of modified Rankin Scale score were 1 (0–3), 2 (1–4), and 6 (5–6) for patients in low-stable, moderate-stable, and persistent-high group, respectively (Fig. 2). Ordinal analysis showed that patients in persistent-high group had increased odds of higher modified Rankin Scale score (adjusted OR 19.06, 95% CI 7.15–50.84), in comparison to patients in low-stable group (Table 3). The corresponding ORs for two extreme tertile comparisons of mean and CV of heart rate were 3.11 (95% CI 1.80–5.37) and 2.08 (95% CI 1.23–3.53).

**Table 3:**
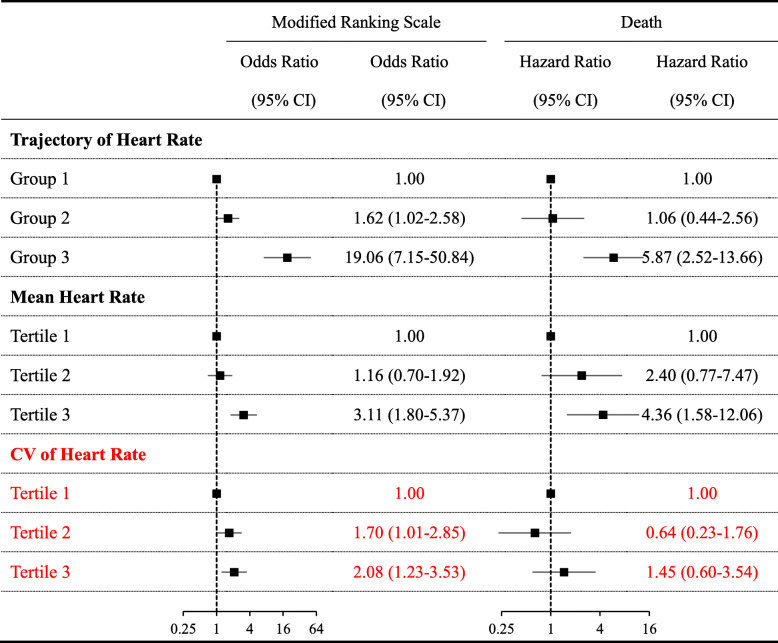
Dynamic changes of heart rate in the acute phase and modified Ranking Scale and death in patients with acute intracerebral hemorrhage

Ordinal logistic regression analyses were used for modified ranking scale. Odds ratios or hazard ratios were adjusted for age, sex, time from onset to admission, current smoking status, systolic BP, fasting plasma glucose, high-density lipoprotein cholesterol, medical history (hypertension, diabetes mellitus, stroke, and atrial fibrillation), types of antihypertensive medications, baseline NIHSS score, location and volume of hematoma

*CI*, confidence interval; *CV*, coefficient of variation

**Fig. 3:**
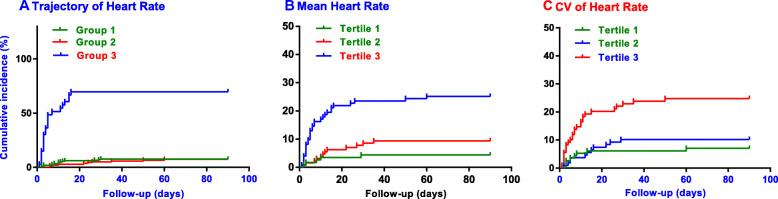
Kaplan-Meier survival curves. Patients in persistent-high trajectory: highest tertile of mean and CV had highest cumulative incidence of 3-month mortality (all log-rank *P* < 0.001). **a** Trajectory of heart rate. **b** Mean heart rate. **c** CV of heart rate

Figure 3 presents that the cumulative incidences of all-cause mortality were highest among participants in persistent-high group or highest mean and CV tertiles of heart rate (all log-rank *P* < 0.001). In the multivariable-adjusted models, the HRs of all-cause mortality were 5.87 (95% CI 2.52–13.66) for persistent-high group, 4.36 (95% CI 1.58–12.06) for highest tertile of mean heart rate, and 1.45 (95% CI 0.60–3.54) for highest tertile of CV, compared with patients in low-stable trajectory or lowest tertile (Table 3).

## Discussion

In this prospective study of patients with acute ICH, we first identified 3 trajectories of patients with a similar pattern of heart rate dynamic change during the acute phase. Patients with persistently high heart rate had the highest risk of unfavorable functional outcome and all-cause mortality at 3 months. These relationships were independent of important confounders, including age, sex, admission systolic BP, medical history, stroke severity, and hematoma volume and location. Moreover, we also found that higher mean and CV of heart rate during the acute phase were independently associated with increased risk 3-month unfavorable functional outcome after ICH. To the best of our knowledge, this is the first study to explore the prognostic value of distinct heart rate trajectories and presented a comprehensive picture of the associations between heart rate dynamic change during the acute phase and clinical outcomes after ICH.

Resting heart rate is a simple and modifiable autonomic parameter and is known as a risk factor of cardiovascular events and mortality in the setting of primary prevention or in several pathophysiologic conditions [22–25]. Moreover, as heart rate may change over time, prior studies also evaluated the predictive effects of longitudinal changes in heart rate [3, 4, 26]. For example, the Atherosclerosis Risk in Communities (ARIC) study indicated that time-updated heart rate and temporal change in heart rate over a median of 3 years were associated with mortality and nonfatal outcomes of incident heart failure, myocardial infarction, and stroke [3]. Sharashova et al. identified five long-term heart rate trajectories using trajectory analysis and found that increasing and elevated trajectories were associated with an increased risk of myocardial infarction and total death in male participants of the Tromsø Study [4]. In another analysis based on 12,554 participants from the Kailuan study, elevated stable heart rate trajectory pattern had the highest risk of having arterial stiffness, an earliest manifestation of adverse structural and functional changes within the vessel wall [26]. These findings suggested that longitudinal changes of heart rate are of great prognostic importance for cardiovascular events and mortality.

Autonomic dysfunction is common after acute stroke and associates with both short-term and long-term adverse stroke outcomes [10–12]. A post hoc analysis of the Prevention Regimen for Effectively Avoiding Second Strokes (PRoFESS) trial reported that higher heart rate at baseline was a risk indicator for mortality and a low heart rate was associated with a better functional outcome and less cognitive decline after ischemic stroke [11]. In addition, emerging evidence from epidemiological studies also suggested that heart rate variability was associated with mortality and functional outcome after acute ischemic stroke [13, 14]. However, few population-based studies have assessed the associations between heart rate and clinical outcomes in patients with ICH. Szabo et al. analyzed the data of 47 ICH patients and found that autonomic parameters evaluated by frequency domain analysis of heart rate variability were associated with stroke severity, intraventricular involvement, and poor outcomes after acute ICH [27]. The pooled analysis of the pilot and main phases of the Intensive Blood Pressure Reduction in Acute Cerebral Hemorrhage Trial (INTERACT) indicated that higher admission heart rate was independently associated with higher mortality and poorer functional outcome after acute ICH [15]. However, a single heart rate measurement might not be sufficient for characterizing the relationship between dynamic change of heart rate during the acute phase and stroke outcomes. The trajectory analysis simultaneously estimates the average, variability, and the direction of variability to investigate the heterogeneity in heart rate trajectories during acute phase and may provide additional information [21]. We found that patients in persistent-high trajectory of heart rate were associated with 15.06-fold increased risk for unfavorable functional outcome and 5.87-fold for all-cause mortality in patients with acute ICH. Consistently with previous studies conducted in acute ischemic stroke patients, we additionally observed that higher level and variability of heart rate in the acute phase was associated with increased risk of unfavorable functional outcome after acute ICH.

Autonomic nervous system dysfunction, including increased sympathetic activity and impaired baroreflex, may underlie the observed relationship of heart rate dynamic change and stroke outcomes [10, 28]. Autonomic dysfunction was implicated in various pathological conditions such as impaired cerebral autoregulation, blood-brain barrier dysfunction, dehydration, hypoglycemia, and systemic inflammation, and then may lead to secondary brain injury and unfavorable clinical outcomes after ICH [28–30].

This study had important clinical implications and highlighted the importance of monitoring heart rate in the acute phase of ICH. The findings suggested that in the acute phase of ICH, maintaining heart rate at an acceptable low level and avoiding repeated fluctuations may contribute to a better prognosis. Both experimental studies and clinical trials indicated that lowering heart rate with existing agents, such as ivabradine, β blockers, and digoxin, may reduce atherosclerotic plaque formation and ischemic injury and may have benefit in relation to cardiovascular events [31–33]. Future clinical trials are warranted to test whether heart rate reduction in the acute phase would improve prognosis of ICH.

The strengths of our prospective study included rigid quality control procedures in the data collection and comprehensive adjustment of important covariates, such as hematoma volume and location, severity of stroke, and medical history. However, our findings should be interpreted in light of some limitations. First, this was an observational study with small sample size, and we could not test causality and exclude the possibility of residual confounding, although we had carefully controlled for multiple stroke-specific risk factors. Second, several complications (e.g., ventilator-associated pneumonia, shock, and fever) may affect both heart rate and outcome, while we did not have these data. Furthermore, we did not collect hematoma growth, mechanical ventilation, and intravascular volume status, which may help to better understand the effects of dynamic changes of heart rate after acute ICH. Third, study participants were from a single center in China, and this may limit the generalizability of our findings to other samples of ICH patients. Finally, we only collected data on all-cause mortality, not cause-specific death due to limited events.

## Conclusions

We found that persistently high heart rate and higher variability in the acute phase were associated with increased risk of unfavorable functional outcome in patients with acute ICH. These findings suggested an important protective role of early heart rate management in the acute phase of ICH. Further prospective studies with larger sample size conducted among different populations with acute ICH are needed to verify our findings.

## Supplementary Information

**Additional file 1: Supplemental Table 1.** Types of antihypertensive medications according to the trajectory of heart rate. **Supplemental Table 2.** Baseline characteristics of patients with acute intracerebral hemorrhage. **Supplemental Figure 1.** Patients flow chart. **Supplemental Figure 2.** The distribution of Posterior predicted probability

## Data Availability

Data are available to researchers on request for purposes of reproducing the results or replicating the procedure by directly contacting the corresponding authors.

## Abbreviations

ICH: Intracerebral hemorrhage
NIHSS: National Institutes of Health Stroke Scale
BP: Blood pressure
CV: Coefficient of variation
BIC: Bayesian information criterion
OR: Odds ratio
HR: Hazard ratio
CI: Confidence interval
