# Supplementary material for: Dynamic change of heart rate in the acute phase and clinical outcomes after intracerebral hemorrhage: a cohort study
Source: J Intensive Care. 2021 Mar 18;9:28. doi: 10.1186/s40560-021-00540-0 (PMC7971394; doi:10.1186/s40560-021-00540-0)
Supplement: Supplementary file 1 — Additional file 1: Supplemental Table 1. Types of antihypertensive medications according to the trajectory of heart rate. Supplemental Table 2. Baseline characteristics of patients with acute intracerebral hemorrhage. Supplemental Figure 1. Patients flow chart. Supplemental Figure 2. The distribution of Posterior predicted probability [file 40560_2021_540_MOESM1_ESM.docx]

Supplemental Table 1. Types of antihypertensive medications according to the trajectory of heart rate.

|  |  | **Trajectory of Heart Rate** | | | |
| --- | --- | --- | --- | --- | --- |
| Characteristics ^a^ | Total population  (n=332) | Group 1: Low-stable (n=174) | Group 2: Moderate-stable (n=126) | Group 3: Persistent-high (n=32) | *P* value |
| **Antihypertensive medications** | |  |  |  | 0.001 |
| None | 112 (33.7) | 72 (41.4) | 29 (23.0) | 11 (34.4) |  |
| Calcium channel blocker | 58 (17.5) | 37 (21.3) | 15 (11.9) | 6 (18.8) |  |
| Beta‐blocker | 0 (0.0) | 0 (0.0) | 0 (0.0) | 0 (0.0) |  |
| ACE inhibitor/ARB | 21 (6.3) | 7 (4.0) | 11 (8.7) | 3 (9.4) |  |
| Diuretic | 3 (0.9) | 1 (0.6) | 1 (0.8) | 1 (3.1) |  |
| Combination ≥2 drugs | 138 (41.6) | 57 (32.8) | 70 (55.6) | 11 (34.4) |  |

Categorical variables are expressed as number (%).

Abbreviation: ACE, angiotensin-converting enzyme; ARB, angiotensin II receptor blockers.

Supplemental Table 2. Baseline characteristics of patients with acute intracerebral hemorrhage.

|  |  | Functional outcome | |  |
| --- | --- | --- | --- | --- |
| Characteristics ^a^ | Total population  (n=332) | Good  (n=229) | Poor  (n=103) | *P* value |
| **Demographics** |  |  |  |  |
| Age, y | 64.3 ± 13.7 | 60.9 ± 13.4 | 71.7 ± 11.3 | <0.001 |
| Male | 224 (67.5) | 160 (69.9) | 64 (62.1) | 0.16 |
| Current smoking | 69 (20.8) | 50 (21.8) | 19 (18.5) | 0.48 |
| **Clinical features** |  |  |  |  |
| Time from onset to admission, h | 5.0 (3.0-24.0) | 6.0 (3.0-24.0) | 4.0 (3.0-7.0) | 0.007 |
| Systolic BP, mm Hg | 169.2 ± 28.9 | 168.6 ± 29.8 | 170.5 ± 27.0 | 0.58 |
| Diastolic BP, mm Hg | 94.9 ± 16.7 | 96.1 ± 17.2 | 92.2 ± 15.1 | 0.05 |
| Triglyceride, mmol/L | 1.0 (0.8-1.4) | 1.1 (0.8-1.5) | 1.0 (0.7-1.3) | 0.07 |
| Total cholesterol, mmol/L | 4.6 (4.1-5.4) | 4.5 (4.0-5.4) | 4.8 (4.2-5.3) | 0.11 |
| LDL-cholesterol, mmol/L | 2.8 (2.3-3.4) | 2.7 (2.3-3.4) | 2.8 (2.3-3.5) | 0.49 |
| HDL-cholesterol, mmol/L | 1.3 (1.1-1.6) | 1.2 (1.0-1.5) | 1.5 (1.1-1.7) | <0.001 |
| Fasting plasma glucose, mol/L | 5.9 (5.2-7.0) | 5.8 (5.2-6.7) | 6.4 (5.6-8.2) | <0.001 |
| Baseline NIHSS score | 8 (3-13) | 5 (2-9) | 15 (12-24) | <0.001 |
| **Medical history** |  |  |  |  |
| History of hypertension | 275 (82.8) | 192 (83.8) | 83 (80.6) | 0.47 |
| History of diabetes mellitus | 41 (12.4) | 25 (10.9) | 16 (15.5) | 0.24 |
| History of stroke | 62 (18.7) | 34 (14.9) | 28 (27.2) | 0.008 |
| History of atrial fibrillation | 9 (2.7) | 4 (1.8) | 5 (4.9) | 0.11 |
| **Antihypertensive medications** | 220 (66.3) | 162 (70.7) | 58 (56.3) | 0.01 |
| **CT findings** |  |  |  |  |
| Hematoma volume (mL) | 10.9 (5.0-23.8) | 9.4 (4.5-19.3) | 18.3 (8.1-43.7) | <0.001 |
| Hematoma location |  |  |  | 0.01 |
| Lobar | 47 (14.2) | 36 (15.7) | 11 (10.7) |  |
| Basal Ganglia | 161 (48.5) | 119 (52.0) | 42 (40.8) |  |
| Thalamus | 21 (6.3) | 13 (5.7) | 8 (7.8) |  |
| Cerebellum | 12 (3.6) | 11 (4.8) | 1 (1.0) |  |
| Brain stem | 28 (8.4) | 16 (7.0) | 12 (11.7) |  |
| Intraventricular extension | 63 (19.0) | 34 (14.9) | 29 (28.2) |  |
| **Trajectory of Heart Rate** |  |  |  | <0.001 |
| Group 1: Low-stable | 174 (52.4) | 138 (60.3) | 36 (35.0) |  |
| Group 2: Moderate-stable | 126 (38.0) | 86 (37.6) | 40 (38.8) |  |
| Group 3: Persistent-high | 32 (9.6) | 5 (2.2) | 27 (26.2) |  |
| **Mean Heart Rate, bpm** | 77 (72-86) | 76 (71-82) | 84 (76-99) | <0.001 |
| **CV of Heart Rate, bpm** | 10.1 (7.4-13.9) | 9.4 (6.9-12.7) | 12.0 (9.1-15.7) | <0.001 |

^a^ Continuous variables are expressed as mean ± standard deviation or median (interquartile range). Categorical variables are expressed as number (%).

Abbreviation: BP, blood pressure; LDL, low density lipoprotein; HDL, high density lipoprotein; NIHSS, National Institute of Health Stroke Scale; CV, coefficient of variation.


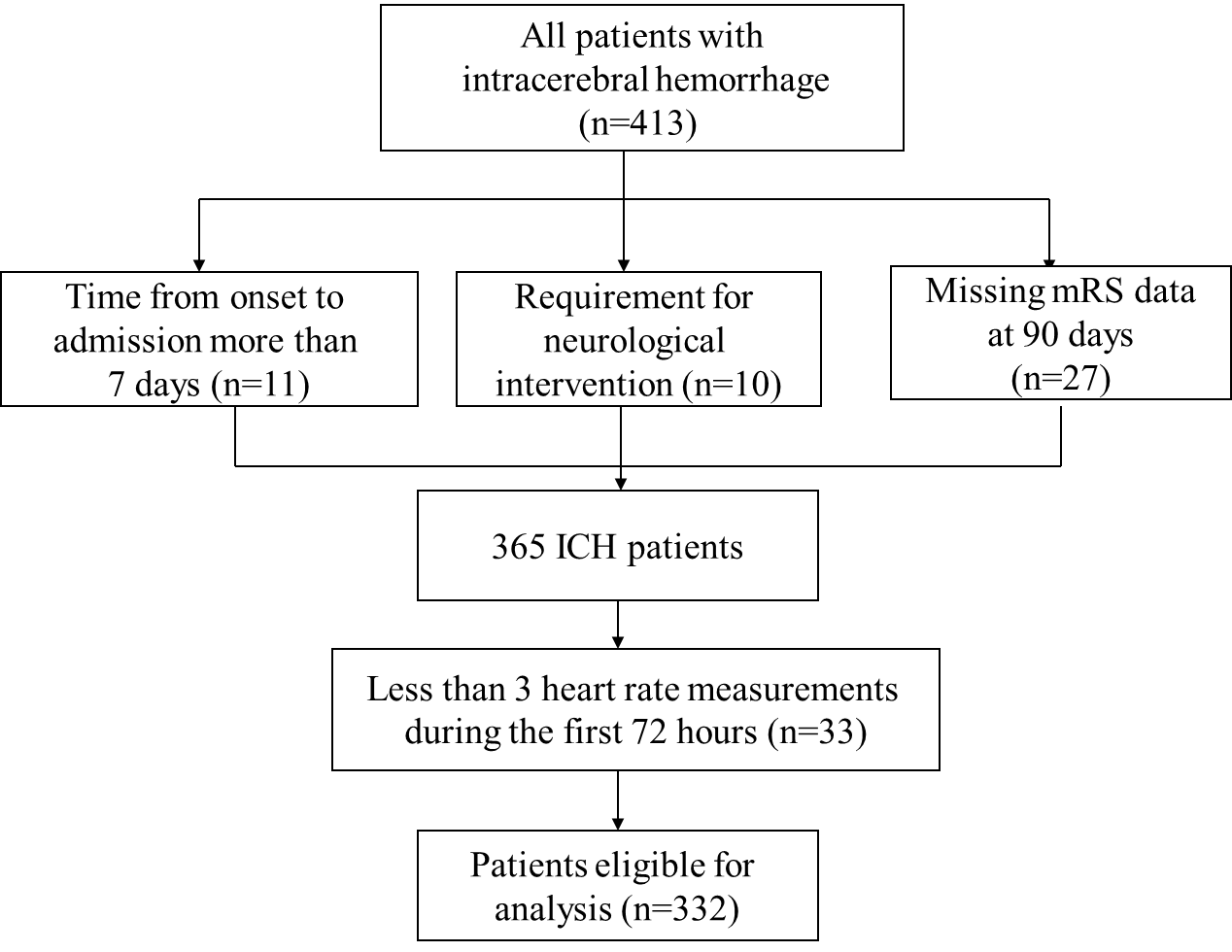


Supplemental Figure 1. Patients flow chart.

Supplemental Figure 2. The distribution of Posterior predicted probability.


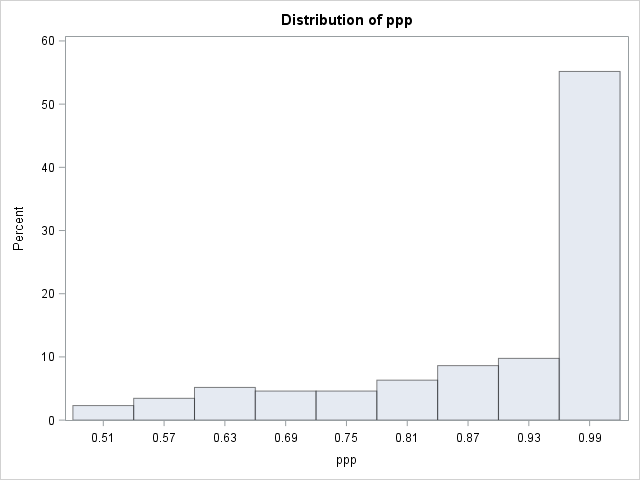


**Distribution of Posterior predicted probability**


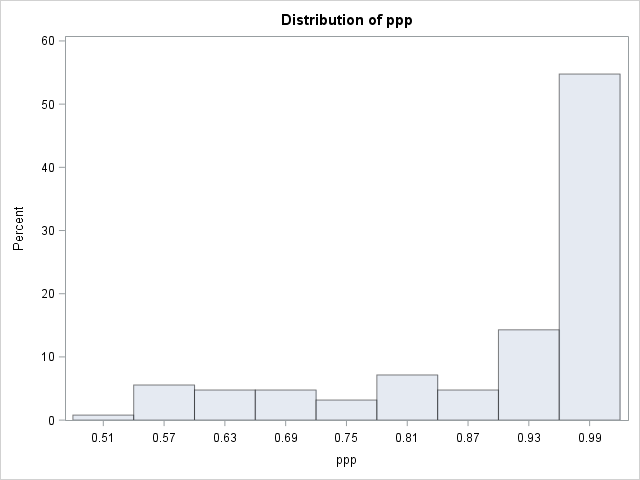

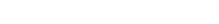

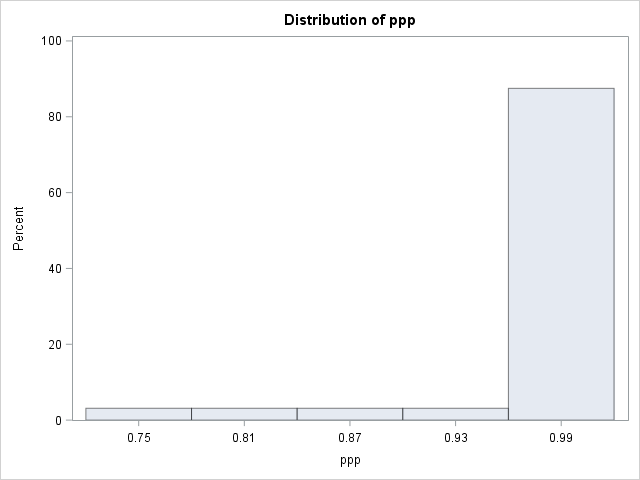

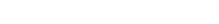


**Posterior predicted probability**

**C**

**B**

**A**

Panel A: Low-stable trajectory; Panel B: Moderate-stable trajectory; Panel C: Persistent-high trajectory.
